# Supplementary material for: Anti-Inflammatory Effects of Auranamide and Patriscabratine—Mechanisms and In Silico Studies
Source: Molecules. 2022 Aug 5;27(15):4992. doi: 10.3390/molecules27154992 (PMC9370761; doi:10.3390/molecules27154992)

Isolation of aurnamide and patriscabratine:

Melastoma malabathricum plant was purchased from a local nursery in Kuala Lumpur, Malaysia. A plant taxonomist confirmed its identity. The voucher specimen was kept in IMU research laboratories for future reference. The leaves were air dried in a hot air oven at a temperature not more than 40°C and powdered using a kitchen blender. The leaf powder (1 kg) was extracted with hexane in a soxhlet extractor to remove the fatty substances. Then, the leaf powder was extracted with methanol in a soxhlet extractor and concentrated at a temperature not more than 50°C in a rotary evaporator (Rotavapor® R-300, BÜCHI Labortechnik AG)under a vacuum. The concentrated extract was completely dried using a freeze dryer. The dried extract was dissolved in sufficient methanol, silica gel 60 was added to form a slurry, and the solvent was evapourated in a rotatory evaporator to obtain a dry homogeneous mixture.

The mixture was transferred to a loader tube and connected to Reveleris™ X2 flash chromatography system. The separation was carried out on Reveleris® amino12g flash Cartridge using a solvent system (water and methanol). The flow rate was maintained at 10 mL/min, and elution of the compounds was detected at 254, 265, and 280 nm. The column was equilibrated for 5 minutes and the total run time was 30 minutes. The eluants that show the absorption peaks were collected. The solvent gradient was initially set to be 1% methanol in water for 1 minute. Then the percentage of the methanol was increased to 100% methanol over 29 minutes. The isocratic elution was maintained whenever the absorption peaks were observed until the entire peak was collected. The presence of the compounds was detected using TLC, and the fractions containing the same compounds were combined. The solvent was evapourated to dryness in a rotary evaporator. The compounds were identified using NMR and LC-MS by comparing the peaks with those reported in the literature.

^{1}H NMR spectrum of patriscabratine:

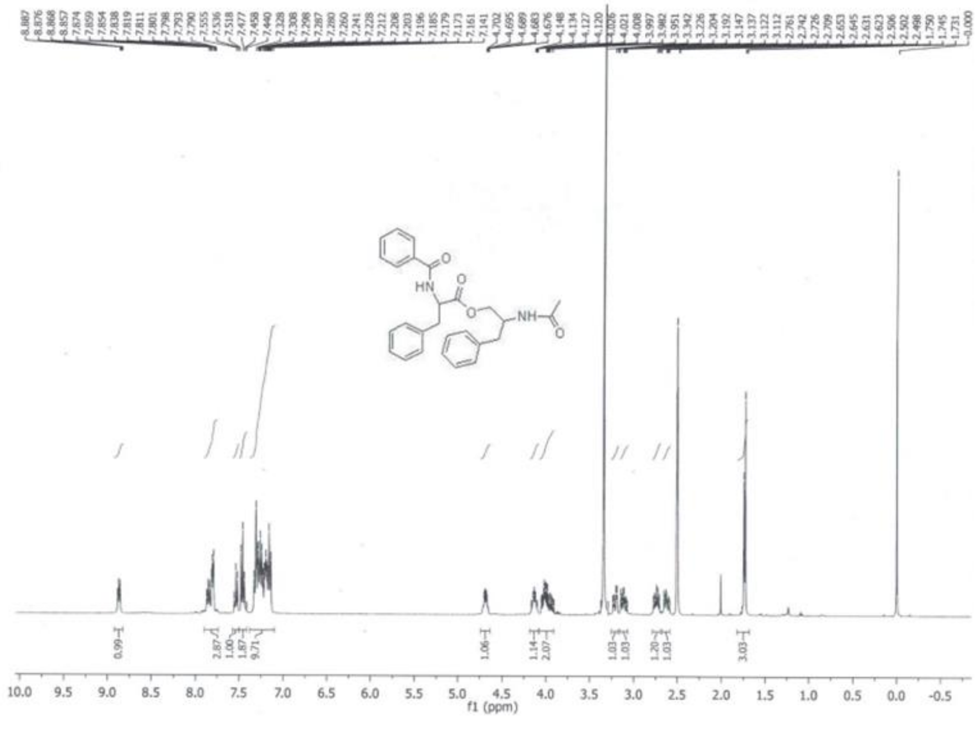

LC-MS spectrum of patriscabratine:

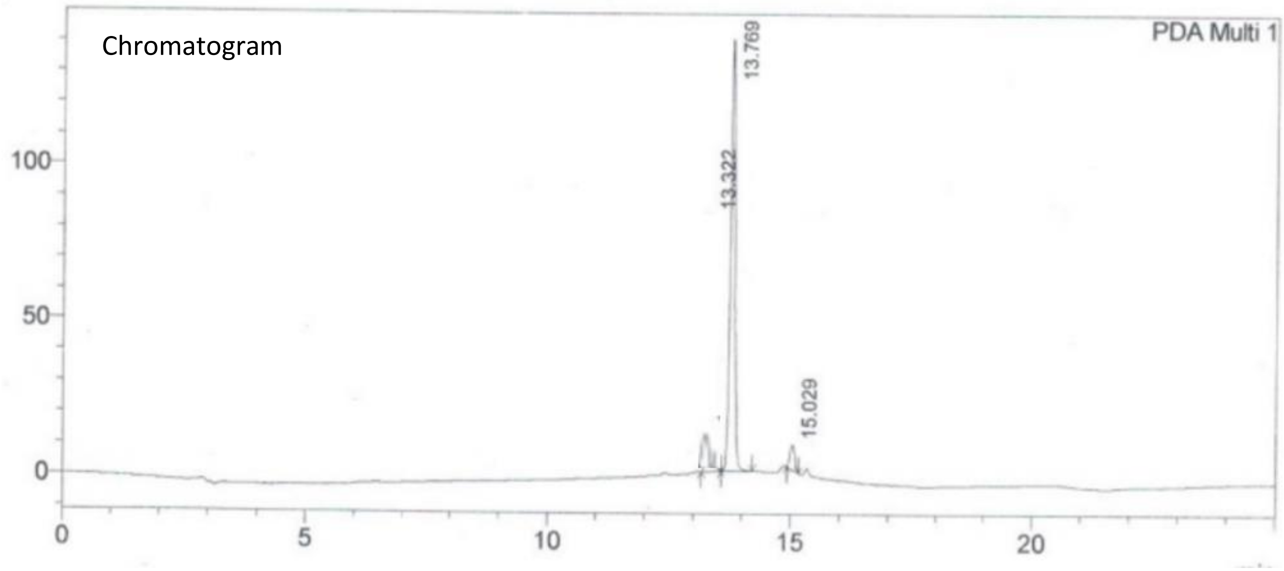
Chromatogram
PDA Multi 1
13.769
13.322
15.029
100
50
0
0
5
10
15
20

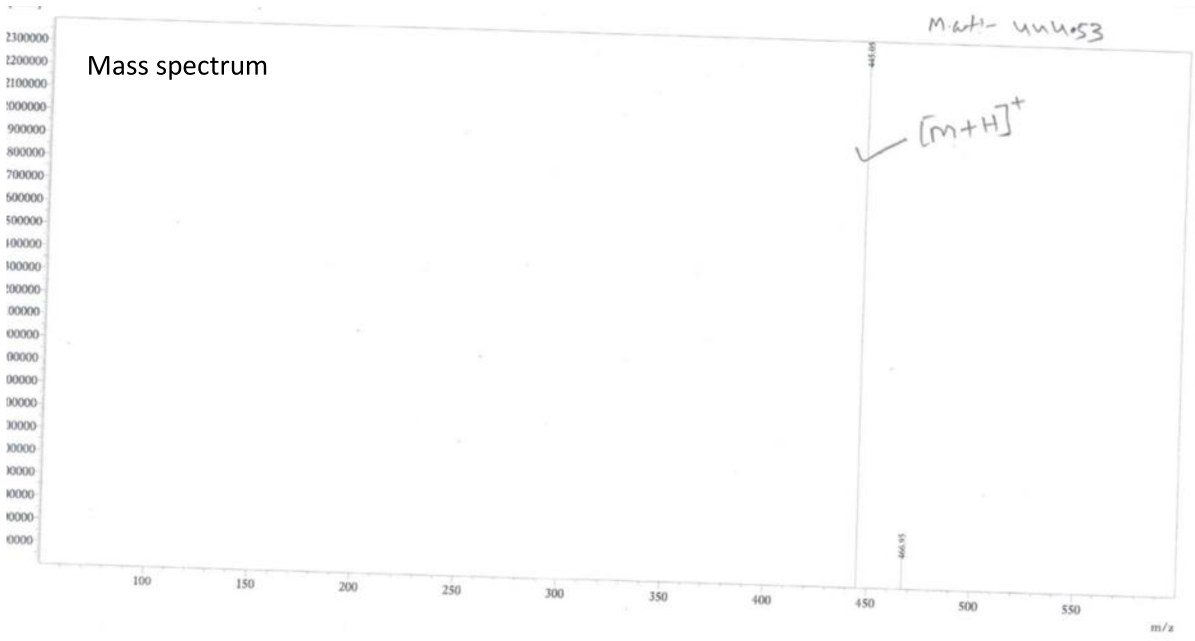
Mass spectrum
M.wt- 444.53
[M+H]+
100
150
200
250
300
350
400
450
500
550
m/z

^{1}H NMR spectrum of auranamide:

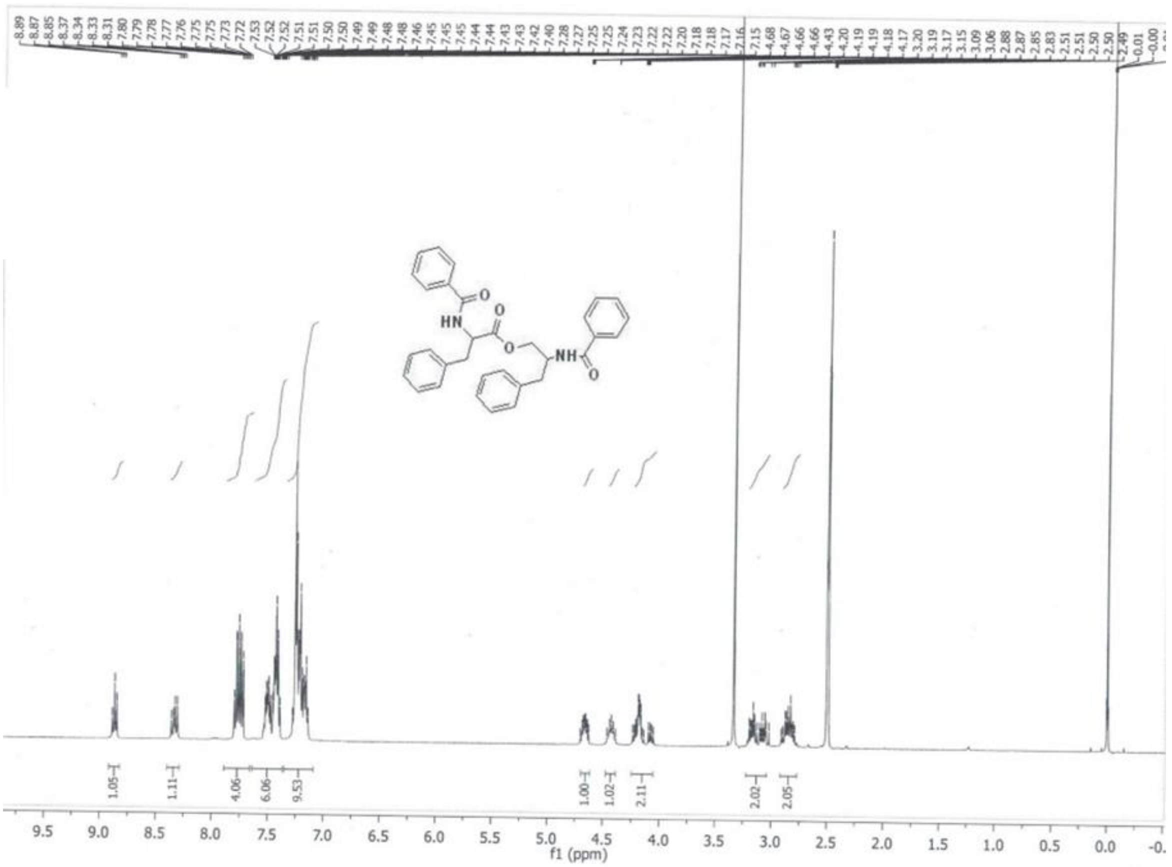

LC-MS spectrum of auranamide:

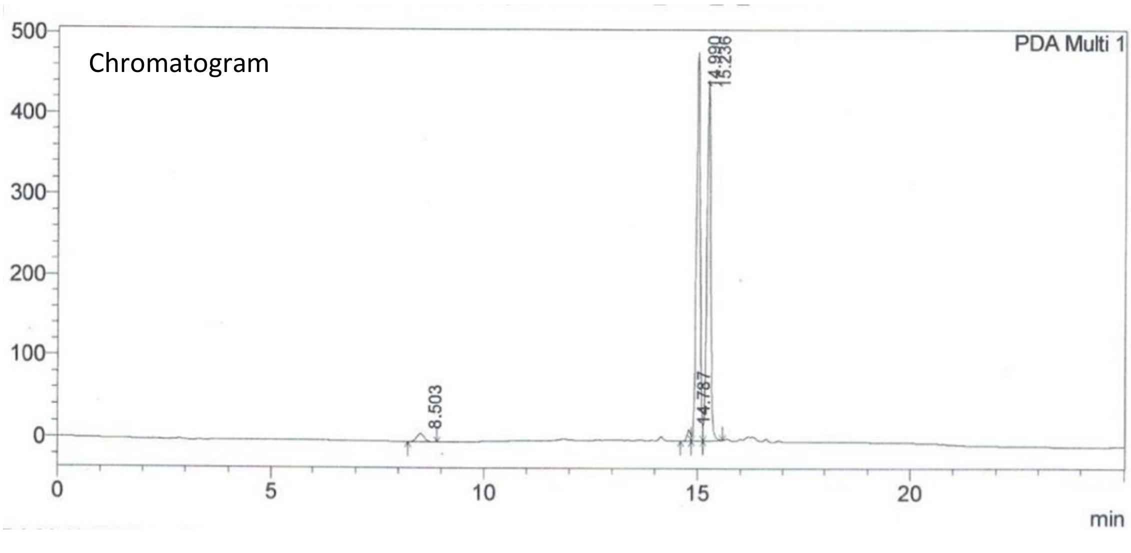

Mass spectrum

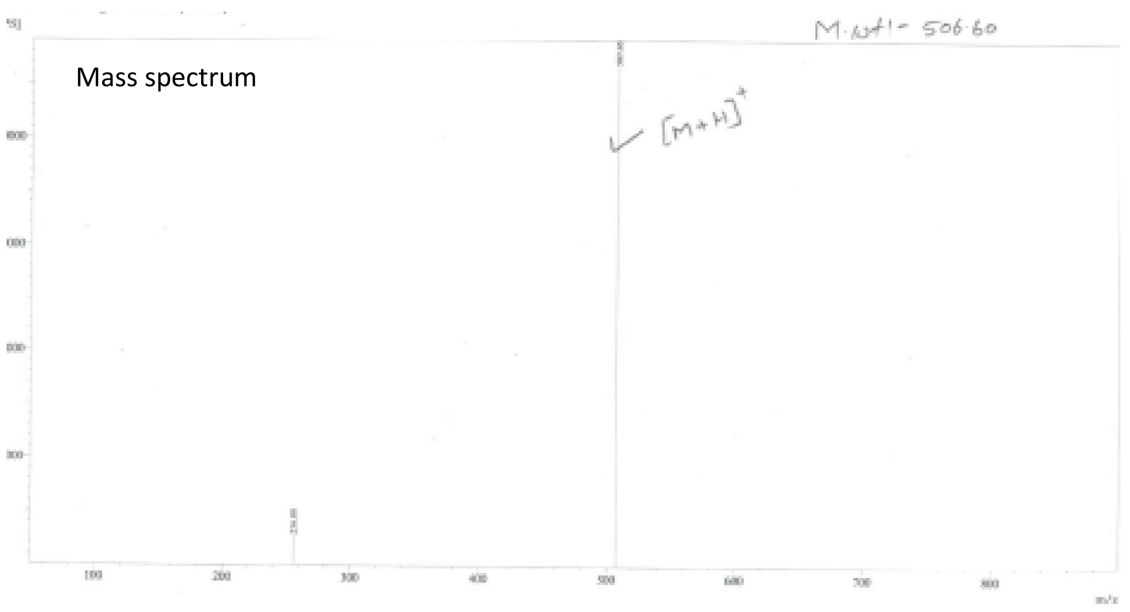

Supplement: Supplementary file 1 [file molecules-27-04992-s001.zip › molecules-1834665-supplementary.pdf]
